# Maximal inter-incisal distance in healthy school children in Switzerland

**DOI:** 10.1186/1546-0096-9-S1-P147

**Published:** 2011-09-14

**Authors:** H van Waes, C Langerweger, L Mueller, CJ Kellenberger, RK Saurenmann

**Affiliations:** 1 Department for Orthodontics and Pediatric Dentistry, University of Zurich, Switzerland; 2 Department for Diagnostic Imaging, Children’s Hospital, University of Zurich, Switzerland; 3 Department for Pediatric Rheumatology, Children’s Hospital; University of Zurich, Switzerland

## Background

The temporo-mandibular joint (TMJ) is affected in about 50% of children with juvenile idiopathic arthritis (JIA). Diagnosis and treatment are often delayed because of lack of symptoms in early TMJ arthritis. A reduced mouth opening capacity may be one of the first clinical signs of TMJ involvement.

## Aim

To create age related percentiles for the maximal mouth opening capacity of healthy children.

## Methods

All recordings of the maximal inter-incisal distance as measured at the yearly routine school dental examinations of school children in the city of Zurich, Switzerland, between August 2009 and August 2010 were extracted from the database. LMS ChartMakerPro version 2.3 (Medical Research Council, UK) was used to calculate age and sex related reference centiles.

## Results

A total of 20’709 measurements (10’058 girls, 10’651 boys) were available for the analysis. The median age (range) was 9.92 years (3.25-18.33) for girls and 10.00 years (2.83-18.67) for boys. The mean inter-incisal distance (range) was 45 mm (25-69) for girls and 45 mm (25-70) for boys. Age related percentiles were created for girls and boys separately, showing the 3^rd^, 10^th^, 25^th^, 50^th^, 75^th^, 90^th^, and 97^th^ percentile from 3 through 18 years of age.

## Conclusion

In these 20’709 unselected school children the inter-incisal distance increased with age but showed a wide range within children of the same age. These percentiles may be a helpful tool in assessing the range of TMJ movement in children with JIA.

